# Folic acid modified TPGS as a novel nano-micelle for delivery of nitidine chloride to improve apoptosis induction in Huh7 human hepatocellular carcinoma

**DOI:** 10.1186/s40360-020-00461-y

**Published:** 2021-01-06

**Authors:** Danni Li, Shaogang Liu, Jiahao Zhu, Liqun Shen, Qi ying Zhang, Hua Zhu

**Affiliations:** 1 School of Chemistry and chemical engineering, Guangxi Key Laboratory for Polysaccharide Materials and Modifications, Guangxi University for nationalities, No.158, Da Xue Xi street, Xixiangtang District, Nanning, 530006 Guangxi Province China; 2 College of Pharmacy, Guangxi University for Chinese medicine, No.13, Wu He street, Qingxiu District, Nanning, 530200 Guangxi Province China

**Keywords:** Nano-micelles, TPGS, Folic acid, Nitidine chloride, Anti-tumor activity, Huh7 human hepatocellular carcinoma line

## Abstract

**Background:**

The development of novel and effective drugs for targeted human hepatocellular carcinoma still remains a great challenge. The alkaloid nitidine chloride (NC), a component of a traditional Chinese medicine, has been shown to have anticancer properties, but doses at therapeutic levels have unacceptable side effects. Here we investigate folic acid modified D-α-tocopheryl polyethylene glycol 1000 succinate (TPGS-FA) as a potential carrier for controlled delivery of the drug.

**Methods:**

Synthesized TPGS-FA was characterized by FTIR, UV-visible and ^1^H NMR spectroscopy, and TPGS loaded with NC was evaluated for its ability to induce apoptosis in Huh7 cells by Annexin V/PI and MTT assays, and observed by laser scanning confocal microscopy and inverted phase contrast microscopy.

**Results:**

TPGS-FA/NC complexes were prepared successfully, and were homogenious with a uniform size of ~ 14 nm diameter. NC was released from the TPGS-FA/NC complexes in a controlled and sustained manner under physiological conditions (pH 7.4). Furthermore, its cytotoxicity to hepatocarcinoma cells was greater than that of free NC.

**Conclusions:**

TPGS-FA is shown to be useful carrier for drugs such as NC, and TPGS-FA/NC could potentially be a potent and safe drug for the treatment of hepatocellular carcinoma.

**Supplementary Information:**

The online version contains supplementary material available at 10.1186/s40360-020-00461-y.

## Background

NC is a benzophenanthridine alkaloid that was initially isolated in 1959 from the roots of Zanthoxylum nitidum (Roxb.) DC (Liangmianzhen in Chinese) [1], a classical traditional Chinese medicinal (TCM) plant found mainly in southern China [2, 3]. Recently, NC was found to show anticancer potential in various human cancer cell lines, including human hepatocellular carcinoma cells [4, 5], although effective therapeutic dosages subsequently revealed hepatotoxicity and kidney damage [6, 7]. The primary liver tumor is hepatocellular carcinoma (HCC) [8–11], but it shows high metastasis and invasion in clinical therapy. However, a high apoptotic capacity has been reported in the early stages of carcinogenesis of HCC cells, although they gradually develop resistance to apoptosis in advanced stages, and the development and progression of HCC is associated with an anti-apoptotic phenotype [12, 13]. Thus novel drugs and effective drug delivery systems for liver cancer therapy need to be developed to overcome this chemoresistance [14].

TPGS, which is a hydrophilic derivative of natural Vitamin E, can be generated by combining polyethylene glycol (PEG) with Vitamin E succinate [15]. TPGS has high permeability and widespread applications as an effective solubilizer, emulsifier, additive, and absorption enhancer [16, 17], and can inhibit the protein of P-gp, which is used as an excipient to overcome multidrug resistance (MDR). More importantly, TPGS has been shown to improve the bioavailability of certain anticancer drugs [18, 19], and has also been reported have selective cytotoxic activity against cancer cells by inducing their apoptosis [20]. Moreover, TPGS-1000 has been approved by the US FDA for use as a drug solubilizer. Optimizing the drug delivery system is a necessary procedure for enhancing the targeting of drugs to tumor cells/tissues [21, 22]. Since a wide range of tumors overexpress folate receptors (FRs) [23, 24], these receptors exhibit high affinity, low immunogenicity, and are potential targets for low cost treatment. Thus, folic acid is commonly used as targeting ligand, which can be easily coupled to various nano-carriers and highly reactive carboxyl groups [25–30]. However, the exact mechanism of the FR delivery system is still not fully understood, although many folate-modified nano-micelles have been discovered with good therapeutic effects, because of receptor-mediated endocytosis [27, 30].

In our present work, we show that it is not always necessary to design a complicated carrier system for drug delivery, and demonstrate that for TPGS-FA/NC dendrimer products have promising applications for use in liver cancer therapy.

## Methods

### Materials

TPGS (PEG = 1000), FA, triethylamine, N-hydroxysuccinimide (NHS), 1-ethyl-3-(3-(dimethylamino)propyl) carbodiimide hydrochloride (EDC), and nitidine chloride (NC) were obtained from the Shanghai Aladdin Biochemical Technology Co. Ltd. (Aladdin, Shanghai, China). Diethylamine was purchased from the Sinopharm Chemical Reagent Co..Ltd. (Aladdin, Shanghai, China). Hoechst33258 was obtained from Shanghai Beyotime Biotechnology Co. Ltd. (Beyotime, Shanghai, China). 5-fluorouracil (5-Fu) was purchased from MedChemExpress (MCE, Monmouth Junction, NJ, USA). Huh7 was purchased from Procell Life Science & Technology Co. Ltd. on July 11, 2019 (identification number: CL-0120, Wuhani, China), Dulbecco’s modified eagle medium (DMEM) was purchased from Life Technologies (AB & Invitrogen) (Gibco, Suzhou, China). Fetal bovine serum (FBS) was purchased from Gemini (Gemini Calabasas, CA, USA). FITC Annexin V apoptosis Detection Kit was purchased from Thermofish Scientific(Thermofish, waltham, USA) MTT was purchased from Beijing Solarbio Science & Technology Co., Ltd.(Solarbio, Beijing, China). Cellulose dialysis membranes (molecular weight cutoff, MWCO = 1000) were acquired from Shanghai Yuanye Biotechnology Corporation (Yuanye, Shanghai, China). Rhodamine B isothiocyanate was acquired from Shanghai Macklin Biochemical Co., Ltd. (Macklin, Shanghai, China). DAPI was obtained from Shanghai Beyotime Biotechnology Co. Ltd. (Beyotime, Shanghai, China). Crystal Violet was purchased from the Beijing Solarbio Science & Technology Co., Ltd. (Solarbio, Beijing, China). Water used in all experiments was purified with a Milli-Q Plus 185 water purification system (Millipore, Thermo Fisher Scientific, US) with resistivity higher than 18.2 MΩ·cm.

### Synthesis of TPGS- FA conjugates

p-Toluenesulfonyl chloride (PTSC) (3.8 g) was added to a solution of TPGS (20 g in 100 mL of CH_2_Cl_2_), followed by dropwise addition of triethylamine (28 mL) under magnetic stirring at room temperature. After 12 h, the reaction mixture was extracted with hydrochloric acid (1 mol/mL) at room temperature. Anhydrous magnesium sulfate was added to the organic phase, which was then stirred, filtered, and concentrated at room temperature. Cold anhydrous ethylether was added dropwise to the condensing filtrate at room temperature, and the white precipitate dried at 40 °C. The TPGS-PTSC complex was obtained by dissolving in 40 mL N, N-dimethylformamide (DMF) under N_2_, then ethylenediamine was added to the TPGS--PTSC solution, which was stirred vigorously for 24 h at 40 °C to produce the TPGS -NH_2_ complex.

**Scheme 1:**
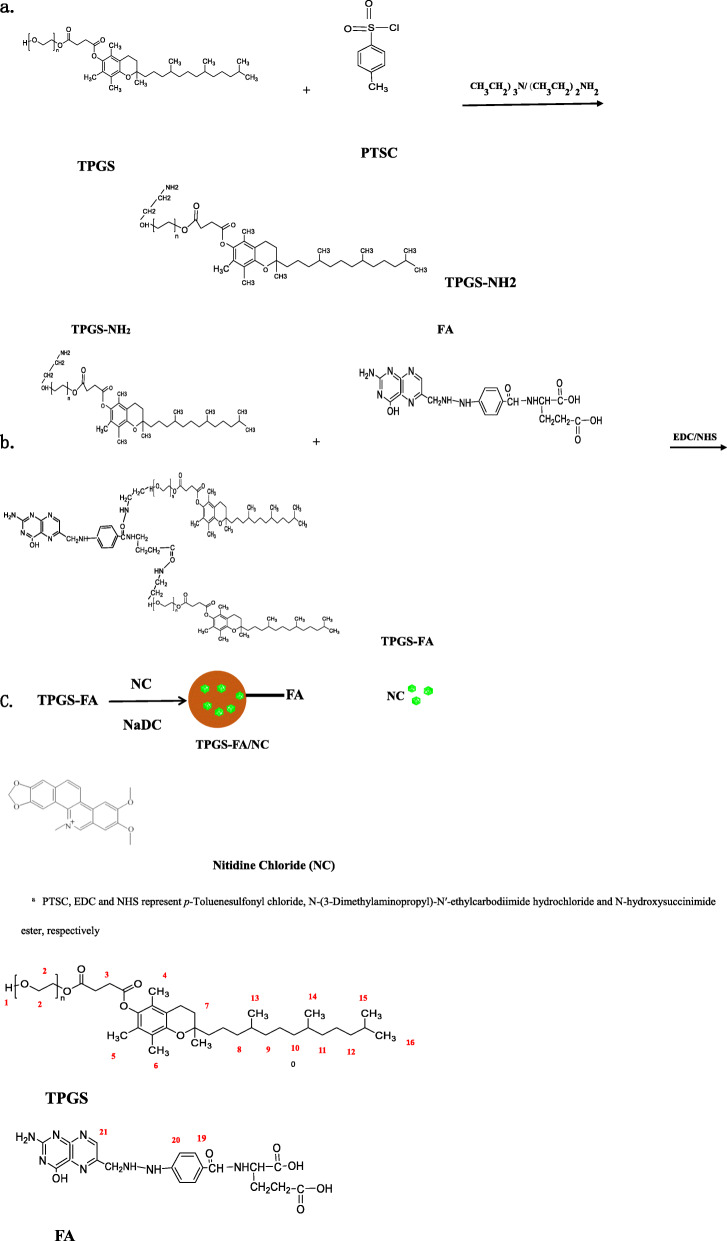
Schematic Illustration of the Preparation of **a** TPGS-NH_2_ Segments, **b** TPGS-FA and **c** the formation of TPGS-FA/NC complexes^a^. ^a^PTSC, EDC and NHS represent *p*-Toluenesulfonyl chloride, N-(3-Dimethylaminopropyl)-N′-ethylcarbodiimide hydrochloride and N-hydroxysuccinimide ester, respectively

FA (44.14 mg) and an equal molar equivalent of EDC (17.14 mg) were dissolved in phosphate buffer (pH 6.0, 0.02 M, 10 mL) stirred vigorously for 0.5 h, followed by dropwise addition of an equal molar equivalent of NHS (10.36 mg). After 3 h, the activated FA solution was added dropwise to a solution of NH_2_-TPGS (100 mg, in 10 mL of phosphate buffer, pH 7.4), and stirred for 3 days at room temperature. The reaction mixture was then dialyzed against water (9 times, 2 L) using a dialysis membrane with an MWCO of 1000 for 3 days to remove any excess reactants. Finally, the sample was lyophilized to obtain a ginger-yellow powder of TPGS-FA (Scheme 1).

### Loading NC into TPGS-FA nano-micelles

Briefly, TPGS- FA (5 mg), NaDC (10.8 mg/mL) and NC (2 mg) were dissolved in phosphate buffer (pH 7.4, 0.02 M, 10 mL) and maintained under vigorous magnetic stirring for 24 h. Then, the complex solution was centrifuged (10,000 rpm for 10 min) to remove the precipitate, which is associated with free NC, and TPGS-FA/NC was obtained from the supernatant by lyophilization. The loading of NC in the nano-micelles was quantified by subtracting the amount of free NC from the initial NC amount. The free NC was quantified by UV absorption at 270 nm in a cuvette (AgilenL Cary60, Agilent Technologies, USA).

### DLS measurement

The size and zeta potential of the nanoparticles in 1 μM deionized water were determined using a Zetasizer Nano-ZS (Malvern ZS90, UK) at 25 °C, and the results plotted using Origin software. Three independent samples were measured for size and zeta potential comparison of TPGS, TPGS-FA/NC, and NC.

### Characterization techniques

^1^H NMR spectra were recorded using nuclear magnetic resonance spectrometer (Bruker AV400, Switzerland). Samples were dissolved in DMSO. Fourier Transform Infrared spectrum (FTIR) were acquired with a MAGNA-1R550 spectrophotometer (Thermo Scientific, USA). Samples were measured as powders in dry potassium bromide.

### In vitro release kinetics

For measuring the release of NC from TPGS-FA/NC complexes, the nano-micelles (1 mg) were dispersed in 1 mL PBS (pH 7.4) or acetate buffer (pH 5.0), placed in a dialysis bag with an MWCO of 8000, and dialyzed against 9 mL of the corresponding buffer medium. All samples were incubated in a constant temperature vibrating bath at 37 °C. At specific time points, 9 mL buffer medium was removed from the outer phase and the concentration of released NC was measured by UV − vis spectroscopy. The volume of the outer phase buffer medium was maintained constant by replenishing with 9 mL of the corresponding buffer solution.. Free NC was also subjected to similar treatment, and used as control. All measurements were performed in triplicate.

### Cell culture

Huh7 cells were regularly cultured in DMEM medium, supplemented with 10% FBS and antibiotics (100 μg/mL streptomycin and 100 μg/mL penicillin) at 37 °C and 5% CO_2_. In all experiments, 5-Fu, TPGS, FA, NC, and TPGS-FA/NC were dissolved in DMSO and diluted in DMEM medium so that the concentration of ethanol was < 1%.

### In vitro apoptosis assay

Cell apoptosis was tested using FITC Annexin V apoptosis Detection Kit. Cells were seeded in six-well plates and cultured overnight. After treatment with 5-Fu, TPGS-FA, NC, or TPGS-FA/NC at a concentration of 50 μg/mL for 24 h, cells were collected and washed with PBS, and then incubated with Annexin V/PI for 15 min. The fluorescence of annexin V/PI was then detected with the BD Accuri ® C6 PLUS flow cytometer (BD Biosciences, USA).

### Confocal microscopy observation

Huh7 cells were seeded at a density of 1 × 10^4^ cells/well in a 6-well culture plate with sterile cover lips. After overnight incubation, the cells attached to the lips. The medium was then replaced with fresh medium containing 5-Fu, TPGS-FA, NC, or TPGS-FA/NC (50 μg/mL) for 24 h. About 3 h later, the cells were fixed with glutaraldehyde (2.5%) for 15 min at 4 °C, then counterstained with Hoechst33258 (1 μg/mL, 1 mL/well) for 15 min at 37 °C, followed by washing 3 times with cold PBS. Finally, the Huh7 cells were imaged using a 20× oil-immersion objective lens and CLSM (Leica SP8, Germany) was applied to determine whether cell apoptosis was induced.

### Evaluation of nano-micelle’ ability to targeted antitumor efficacy in cells

To detect the uptake and internalization of nano-micelles into the cancer cells, the nano-micelles were labeled with rhodamine B isothiocyanate. The rhodamine B isothiocyanate-loaded nano-micelles were prepared by direct dissolution method [31]. Briefly, Rhodamine B isothiocyanate, together with an appropriate amount of the TPGS-FA/NC was dissolved in PBS, and after 30 min of sonication, the solution was maintained at room temperature for further 30 min. The unloaded rhodamine B isothiocyanate was removed by centrifugation and washed in PBS. After 4 h of treatment, the outer fresh medium of the cells labeled with rhodamine B isothiocyanate nano-micelles were removed. After washing twice with PBS buffer, cells were fixed with 4% formaldehyde (15 min) and washed twice with PBS buffer again, followed by treatment with After rinsing with PBS buffer, the cells were mounted with DAPI for cell nucleus staining and assayed on CLSM (Leica SP8, Germany).

### Cytotoxicity assay and cell morphology observation

Huh7 cells were plated onto a 96-well plate at a density of 5000 cells/200 μL per well, and left overnight. They were then treated with 5-Fu, free NC, TPGS, or TPGS-FA/NC (at concentrations of 7.5, 15, 30, 60, or 120 μg/mL), or DMSO for 24 h. 10 μL MTT reagents were added to each well, and untreated cells served as a control. The absorbance (Am) was measured at 490 nm using a microplate reader (Thermofish, USA). The morphology of cells was observed using a inverted phase contrast microscope (Olympus, China) with a magnification of 40× after treatment with 5-Fu, free NC, TPGS, or TPGS-FA/NC nano-micelles, for 24 h.

## Results

### FTIR characterization of TPGS-FA

**Fig. 1:**
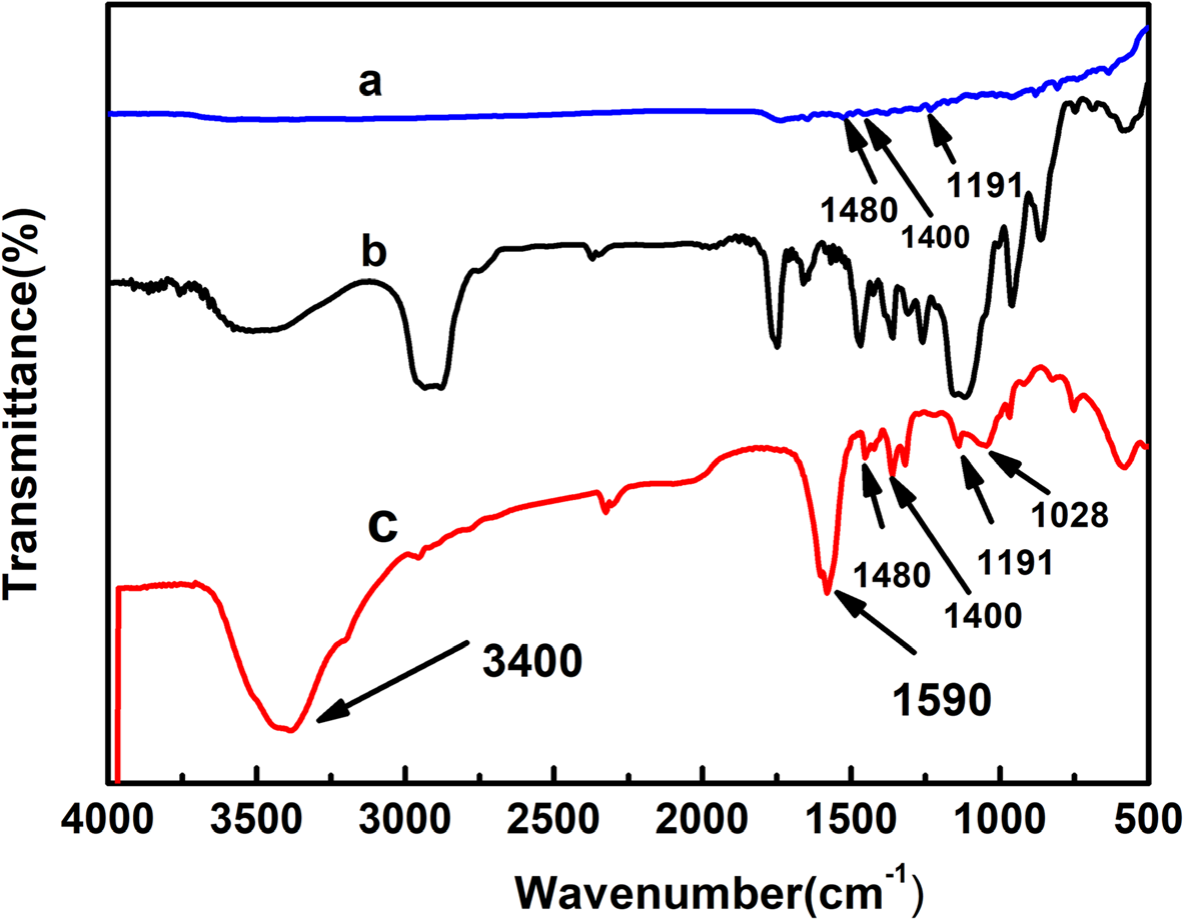
FTIR spectra of **a** FA, **b** TPGS, and **c** TPGS-FA

FTIR was used to demonstrate the successful preparation of TPGS-FA as shown in Fig. 1. The peaks at 1028 cm^− 1^, 1590 cm^− 1^, and 3400 cm^− 1^ are characteristic vibrations of C=O and N-H, respectively, and thus provide evidence for the formation of the amide bond, and demonstrate that FA and TPGS were successfully combined.

### Characterization of the TPGS-FA conjugates

**Fig. 2:**
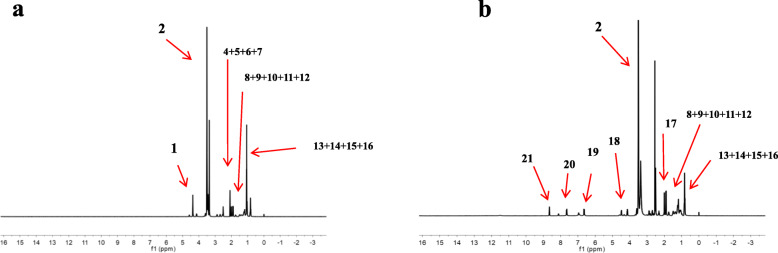
^1^H NMR spectra of **a** TPGS and **b** TPGS-FA. The numbers refer to the positions of the C atoms in the structures shown above

The ^1^H NMR spectrum of TPGS-FA (Fig. 2b) shows that the FA molecule was successfully grafted onto TPGS, and the FA modified TPGS nano-micelles showed δ values at 6.83 ppm (peak 19), 7.65 ppm (peak 20) and 8.64 ppm (peak 21) from the pterin ring of folic acid. The final acetylation of the remaining terminal amines of TPGS-NH_2_ led to the formation of TPGS-FA, and the emergence of a peak at 4.35 ppm (peak 18) is associated with −CH_2_ protons of the acetyl groups, thus confirming the success of the acetylation reaction. By integrating the proton signals associated with the FA moieties and the dendrimer methylene groups, the average number of FA moieties attached onto each dendrimer was estimated to be 1.3. Sch1

**Fig. 3:**
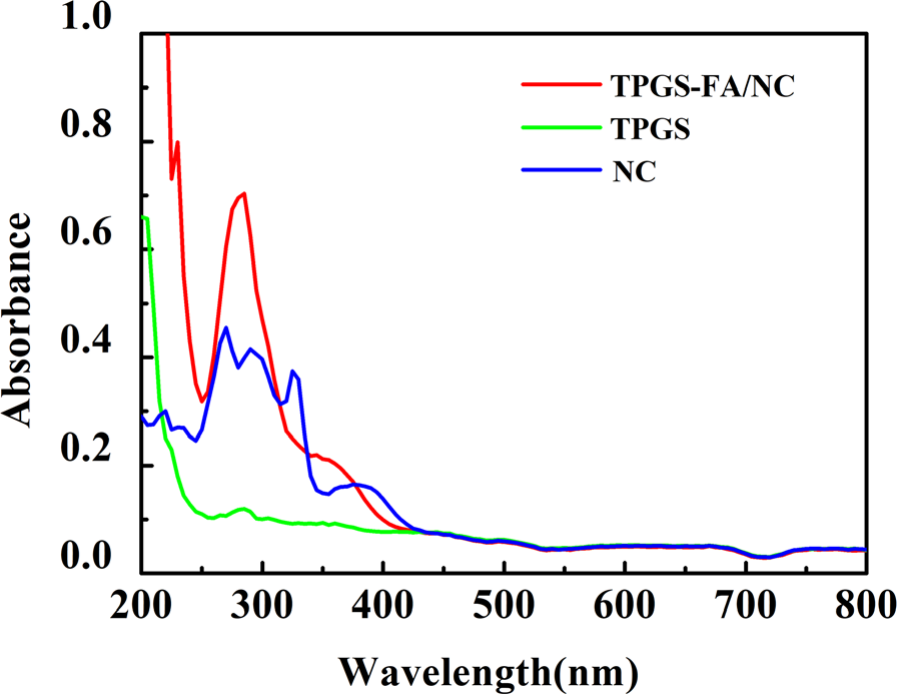
UV − vis spectra of free NC dissolved in ethanol, TPGS, and TPGS-FA/NC dispersed in PBS

UV − vis spectrometry was also used to provide further qualitative confirmation of the successful conjugation of FA onto the TPGS dendrimers (Fig. 3), which is demonstrated by the strong absorption peak at 280 nm in the TPGA-FA/NC dendrimers.

### Encapsulation of NC within the TPGS-FA/NC nano-micelles

The formation of TPGS-FA/NC nano-micelles was demonstrated by UV-visible spectroscopy (Fig. 3), which showed greatly enhanced intensity for the absorption at 270 nm compared to free NC dissolved in ethanol. Quantification using a standard NC absorbance/concentration calibration curve (Y = 0.949X-0.0079, *R*^*2*^ = 0.9995), indicated that an average of 5.2 molecules of NC were encapsulated within each TPGS-FA/NC dendrimer.

### Size and zeta potential

**Fig. 4:**
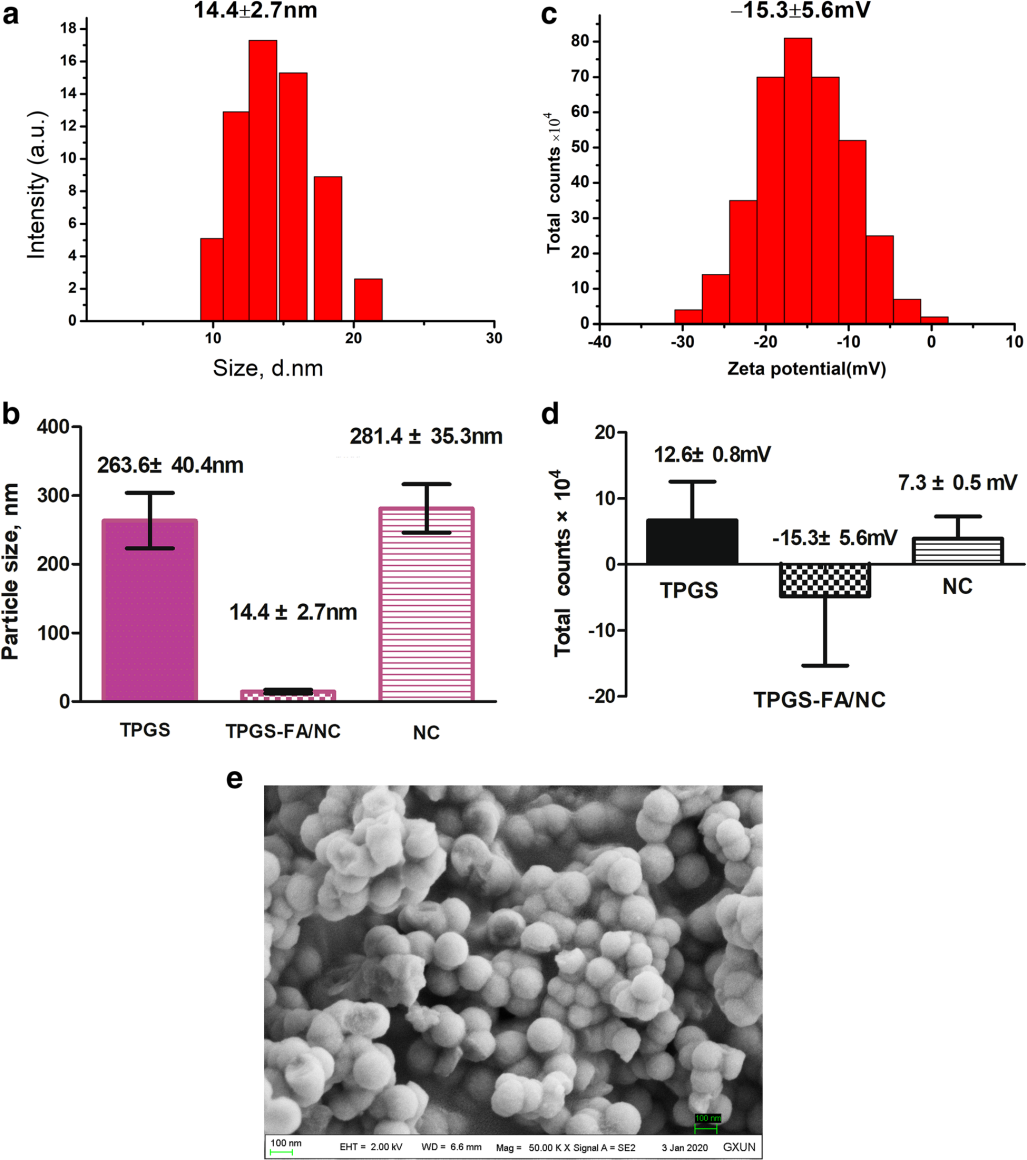
**a** Sizes of TPGS-FA/NC, **b** Size comparison of TPGS, TPGS-FA/NC, and NC (*n* = 3 independent samples, as a Source Data file.) (mean ± SD of one size distribution)., **c** Zeta potential of TPGS-FA/NC (pink), and **d** Zeta potential comparison of TPGS, TPGS-FA/NC, and NC (*n* = 3 independent samples, as a Source Data file) (mean ± SD, as a Source Data file), **e** SEM image of TPGS-FA/NC on Field Emission Scanning Electron Microscopy SUPRA 55 (carl ZEISS Corp, Germany)

DLS measurements of TPGS-FA/NC showed an average hydrodynamic diameter of 14.4 ± 2.7 nm (*n* = 3 independent samples, mean ± SD) (Fig. 4a, Table 1). The low size variability demonstrates that there is an essentially homogeneous assembly of TPGS-FA/NC nano-micelles, and suggests that the TPGS-FA nano-micelles incorporated NC from the aqueous solution, because free NC in aqueous solution is heterogeneous with large size variation (Fig. 4b, Table 1). The zeta potential of the TPGS-FA/NC nano-micelles was − 15.3 ± 5.6 mV (mean ± SD of the zeta potential distribution) (Fig. 4c, Table 1).

**Table 1:** Zeta Potentials and sizes of TPGS, TPGS-FA/NC and NC Complexes^a^

	Zeta potential (mV)	Size (nm)
TPGS	12.6 ± 0.8	263.6 ± 40.4
TPGS-FA/NC	−15.3 ± 5.6	14.4 ± 2.7
NC	7.3 ± 0. 5	281.4 ± 35.3

^a^Data are provided as mean ± S.D.

### In vitro release kinetics

**Fig. 5:**
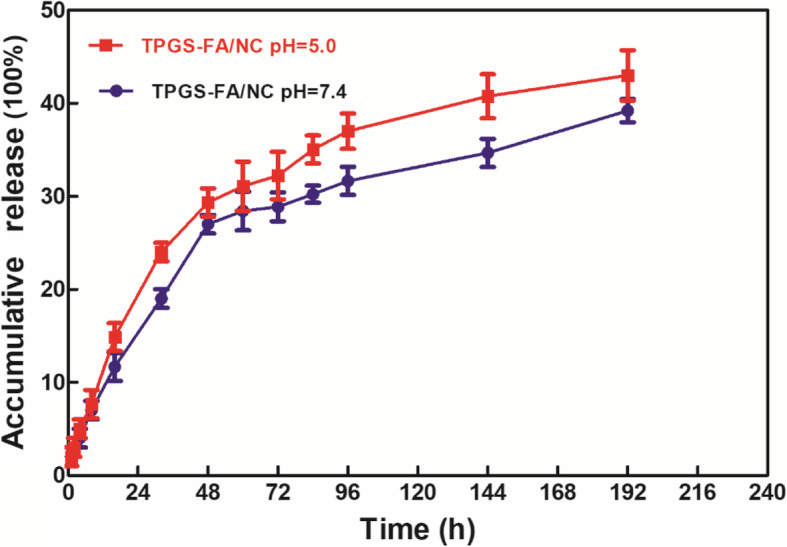
Cumulative release of NC from TPGS-FA/NC complexes in PBS (pH 7.4) and acetate buffer (pH 5.0) at 37 °C

PBS (pH 7.4) and acetate buffer (pH 5.0) were selected as media for evaluating in vitro the kinetics for release of NC from the TPGS-FA/NC complexes at 37 °C (Fig. 5). As shown in Fig. 5, the NC was released progressively over a period of several days, thus demonstrating that the relatively hydrophobic interior of the dendrimer prevents the drug from being released in a rapid burst. Specifically, TPGS-FA/NC complexes showed a relatively slow rate of release of NC, amounting to 39.2 ± 1.3% at pH 7.4 and 43.0 ± 2.7% at pH 5.0 in 192 h. This suggests that a faster release of NC from the TPGS-FA dendrimers occurs when the hydroxyl groups of the TPGS can interact with the encapsulated NC drug via hydrogen bonding and promoting the NC release. The release rate of NC from both dendrimer/NC complexes was faster under acidic condition (pH 5.0) than under physiological conditions (pH 7.4). Under acidic conditions (pH 5.0), there is repulsion between protonated dendrimers and positively charged NC molecules, which increases the rate of release of NC from the interior of the dendrimers. The release kinetics of the TPGS-FA/NC nano-micelles followed a similar trend with respect to the impact of the TPGS-FA and the pH responsive NC release behavior. In contrast, free NC was released rapidly under both pH conditions, and at 1 h, around 81.7 and 73.5% NC were released at pH 5.0 and pH 7.4, respectively.

### TPGS-FA/NC induced hepatocellular carcinoma cell apoptosis

**Fig. 6:**
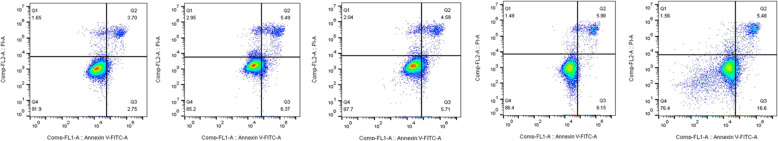
Flow cytometry plots of control Huh7 cells and the groups treated with 5-FU, TPGS-FA, NC, and TPGS-FA/NC at a concentration of 50 μg/mL for 24 h. In vitro apoptosis was observed by propidium iodide (PI)/Annexin V-FITC dual staining and fluorescence-activated cell sorting (FACS) analysis (Q2 = Annexin V-FITC and PI positive, indicating cells in late apoptosis or already dead; Q3 = PI negative & Annexin V-FITC positive, indicating early apoptotic cells). Source data are provided as a Source Data file

**Fig. 7:**
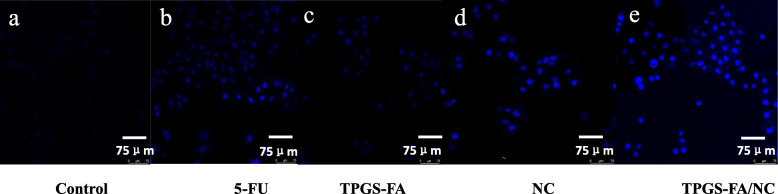
CLSM images of Huh7 cells treated with **a** control, **b** 5-FU, **c** TPGS-FA, **d** NC, and **e** TPGS-FA/NC at a concentration of 50 μg/mL for 24 h, respectively. Scale bar: 75 μm for original images

The in vitro cell apoptosis assay showed that a higher proportion (22.7%) of the cells underwent apoptosis after a 24 h treatment with TPGS-FA/NC nano-micelles compared to treatments with 5-Fu (11.9%), TPGA-FA (10.3%), free NC (12.1%), and the control (6.4%) (Fig. 6). Furthermore, induction of higher levels of cell apoptosis by the TPGS-FA/NC treatment was confirmed by CLSM imaging, and Fig. 7 shows a comparison of the control with 5-Fu (Fig. 7b), TPGS-FA (Fig. 7c), free NC (Fig. 7d), and TPGS-FA/NC (Fig. 7e) after 24 h incubation. It should be noted that the treated samples display significant blue signals, which are associated with the induction of cell apoptosis, and as with the flow cytometric analysis, the fluorescence signals are much stronger than those of cells treated with free NC. Thus these results, highlight the potential of TPGS-FA nano micelles for significantly improving the therapeutic efficacy of NC.

### Targeting specificity of the TPGS-FA/NC

**Fig. 8:**
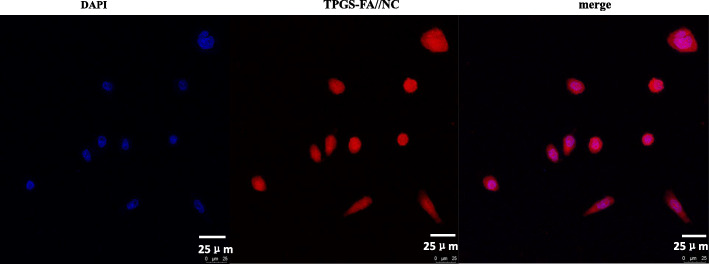
In vitro cell binding of TPGS-FA//NC nano-micelle, shown by confocal microscopy (blue: nucleus; red: TPGS-FA//NC nano-micelle. Scale bar: 25 μm for original images)

The FA-mediated targeting specificity of the TPGS dendrimer was revealed by CLSM imaging of Huh7 cells (Fig. 8, Supporting Information). The confocal microscopic images of the cells treated with rhodamine B isothiocyanate-loaded nano-micelles demonstrated that the nano-micelles were able to enter the Huh7 cells.

### Therapeutic efficacy of the TPGS-FA/NC nano-micelles

**Fig. 9:**
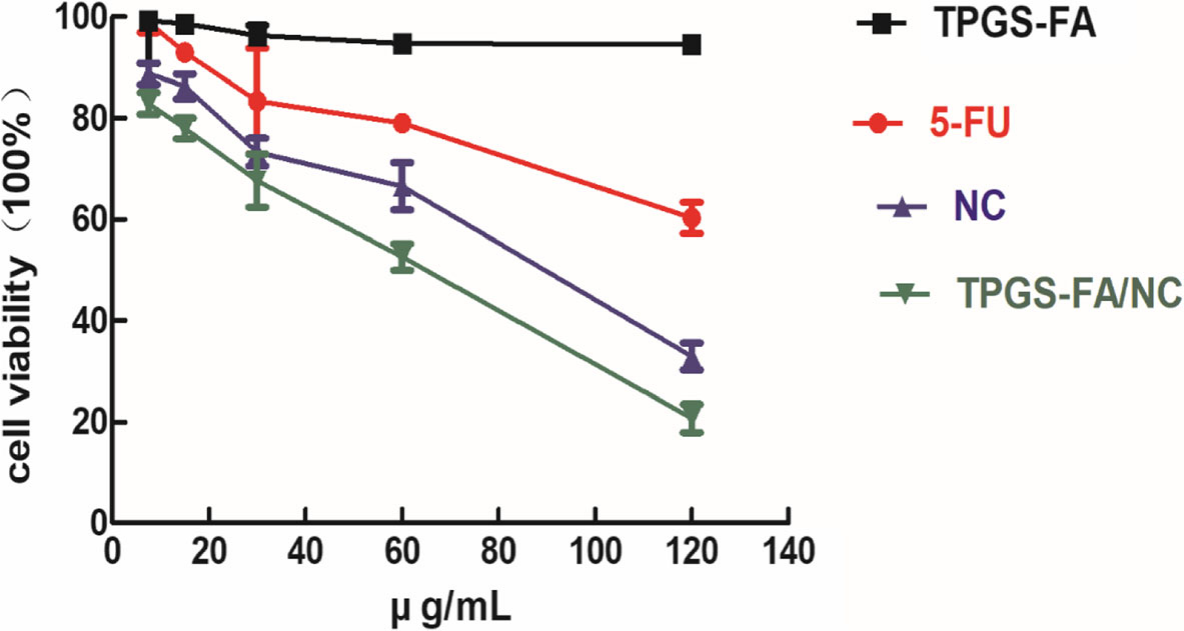
MTT assay of Huh7 cells treated with 5-FU, TPGS-FA, TPGS-FA/NC, and free NC at 7.5, 15,30, 60 and 120 μg/mL for 24 h. The data are expressed as mean ± S. D (n = 3)

To evaluate cytotoxicity, the MTT assay was performed to determine cell viability after treatments with 5-Fu, TPGS-FA, free NC, and TPGS-FA/NC in concentrations of 7.5, 15, 30, 60, or 120 μg/mL) (Fig. 9). Treatment with TPGS-FA/NC nano-micelles significantly inhibited the growth of Huh7 cells with concentrations at or above 30 μg/mL, whereas NC without TPGS-FA showed weaker cytotoxicity, and TPGS-FA without NC had very little effect on cell growth.

**Fig. 10:**
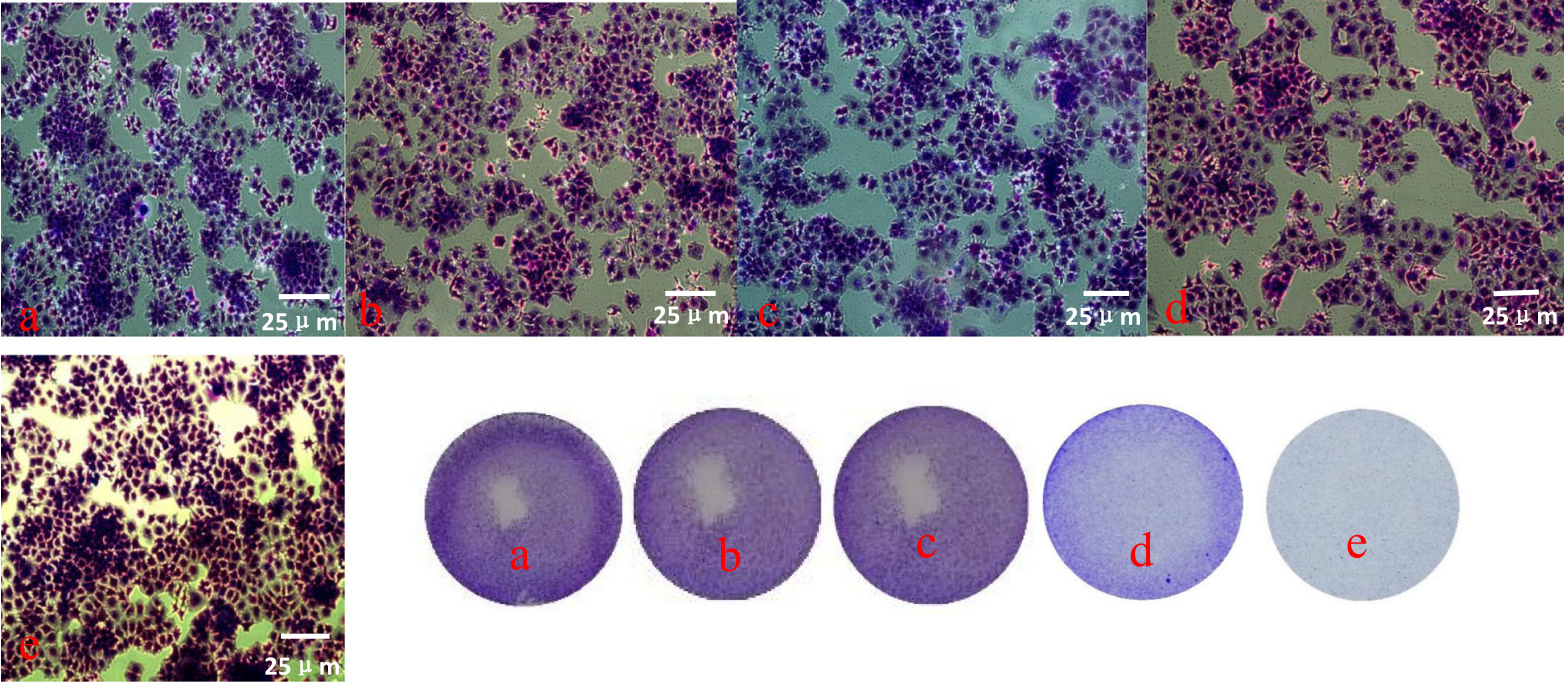
Phase contrast photomicrographs obtained by Crystal Violet staining of Huh7 cells treated with **a** control, **b** 5-FU, **c** TPGS-FA, **d** NC, and **e** TPGS-FA/NC at a concentration of 50 μg/mL for 24 h (Scale bar: 25 μm for original images)

The induction of apoptosis by TPGS-FA/NC micelles was further confirmed by microscopic visualization of the morphology of Huh7 cells. Those treated with TPGS-FA (Fig. 10c) were similar to the control cells treated with PBS (Fig. 10a), indicating that the nano-micelles without NC had little toxicity to the Huh7 cells. However, as shown in Fig. 10e, a significant proportion of the Huh7 cells treated with the TPGS-FA /NC for 24 h became rounded and detached, thus indicating cell death. These results demonstrate that the therapeutic effect of the nano-micelles is associated with the loaded NC, and that encapsulation of NC within the nano-micelles does not compromise its therapeutic efficacy.

## Discussion

In summary, we have made use of the negatively charged TPGS-FA to load and protect the positively-charged NC molecules. However,. TPGS-FA/NC nanomicelles are negatively charged and 14.4 ± 2.7 nm TPGS-FA/NC, which making them have minimal interaction with negatively charged cell membranes. Nanomicelles could limit their capacity of passing through cell membranes. The folic acid could also be used as a targeting ligand, which could help TPGS-FA/NC conjugates specifically enter Huh7 cells via the folate receptor-mediated endocytic pathway. However, both the MTT assay and flow cytometry indicate that TPGS-FA/NC treatment was effective for inducing cell apoptosis, and the associated change in the morphology of Huh7 cells was observed and verified by laser confocal microscopy. It is interesting that TPGS-FA/NC showed higher therapeutic activity than free NC in vitro, and it is possible that the FA-modified nano-micelles exert increased toxicity to Huh7 cells by inhibiting additional biochemical pathways that lead to cell apoptosis.

## Conclusions

Encapsulation of hydrophobic drugs such as NC can improve their water solubility and increase their bioavailability, and our present research shows that TPGS-FA dendrimers may serve as an effective carrier system for sustained release of NC (pH 7.4), tumor-specific targeting, and for application in liver cancer therapy.

## Supplementary Information

**Additional file 1.**

## Data Availability

All data generated or analyzed during the present study are included in this article.

## Abbreviations

FA: Folic acid
TPGS: D-α-tocopheryl polyethylene glycol 1000 succinate
NC: Nitidine chloride
HCC: Hepatocellular carcinoma
PTSC: *p*-toluenesulfonyl chloride
NHS: N-hydroxysuccinimide
EDC: 1-ethyl-3-(3-(dimethylamino)propyl) carbodiimide hydrochloride
DMF: N, N-dimethylformamide
NaDC: Sodium deoxyocholate
DMSO: Dimethyl sulfoxide
DMEM: Dulbecco’s modified eagle medium
FBS: Fetal bovine serum
MTT: 3-(4,5-dimethylthiazol-2-yl)-2,5-diphenyltetrazolium bromide
5-Fu: 5-fluorouracil
