# Supplementary material for: Folic acid modified TPGS as a novel nano-micelle for delivery of nitidine chloride to improve apoptosis induction in Huh7 human hepatocellular carcinoma
Source: BMC Pharmacol Toxicol. 2021 Jan 6;22:1. doi: 10.1186/s40360-020-00461-y (PMC7789002; doi:10.1186/s40360-020-00461-y)

HuH-7 cells STR report

Method: An appropriate amount of HuH-7 cells (Cell number PC156,1×106) was used to extract DNA, 20 STR loci and gender identification loci were amplified by 21 CELLID System, PCR product detection was performed by ABI3130x1 genetic analyzer, detection results were analyzed by GeneMapper IDX software (Applied Biosystems), and compared with ATCC, DSMZ, JCRB, Cellosaurus and other databases.

Experimental result:

1. The results of negative and positive control were correct.
2. The genotyping results of STR locus of HuH-7 cell line are shown in the following table.

Conclusion:

1. The genomic DNA of HuH-7 cell line is clear and the result of genotyping is good.
2. The results of STR typing showed that no cross contamination of mouse cells and human cells was found in the cell line of HuH-7 cell line.
3. The DNA typing of the cell line was 98.18% matched with the cell type in the cell bank, and the cell line name was HuH-7.

Appendix I: The genotyping results of STR locus of HuH-7 cell line

STR Loci	Sample: PC156	Database: HuH-7
Amelogenin	X	X
CSF1PO	11	11
D2S1338	19	19
D3S1358	15	15
D5S818	12	12
D7S820	11	11
D8S1179	14,15	14,15
D13S317	10,11	10,11
D16S539	10	10
D18S51	15	15
D19S433	13,14	13,14
D21S11	30	30
FGA	22	22,23
PentaD	12	12
PentaE	11	11
TH01	7	7
TPOX	8,11	8,11
vWA	16,18	16,18
D1S1656	16	
D6S1043	13,15	
D12S391	20,21	
D2S411		
The Cellosaurus database has a matching rate of 98.18%, The number of matched bits is 20 (https://web.expasy.org/cellosaurus-str-search/)		

Note:

1. According to the cell STR identification standard established by the International Cell Line Authentication Committee (ICLAC), when the matching degree of cell lines is ≥80%, they are considered to be correlated, that is, derived from common ancestral cells; The matching degree is between 55% and 80%, and the correlation needs to be further verified. Less than 55% indicates no correlation between the two.
2. The effective peak of the map was the real PCR band; Small peaks and nonspecific bands were ignored in the calculation.

Appendix III: The genotyping results of STR locus of HuH-7 cell line

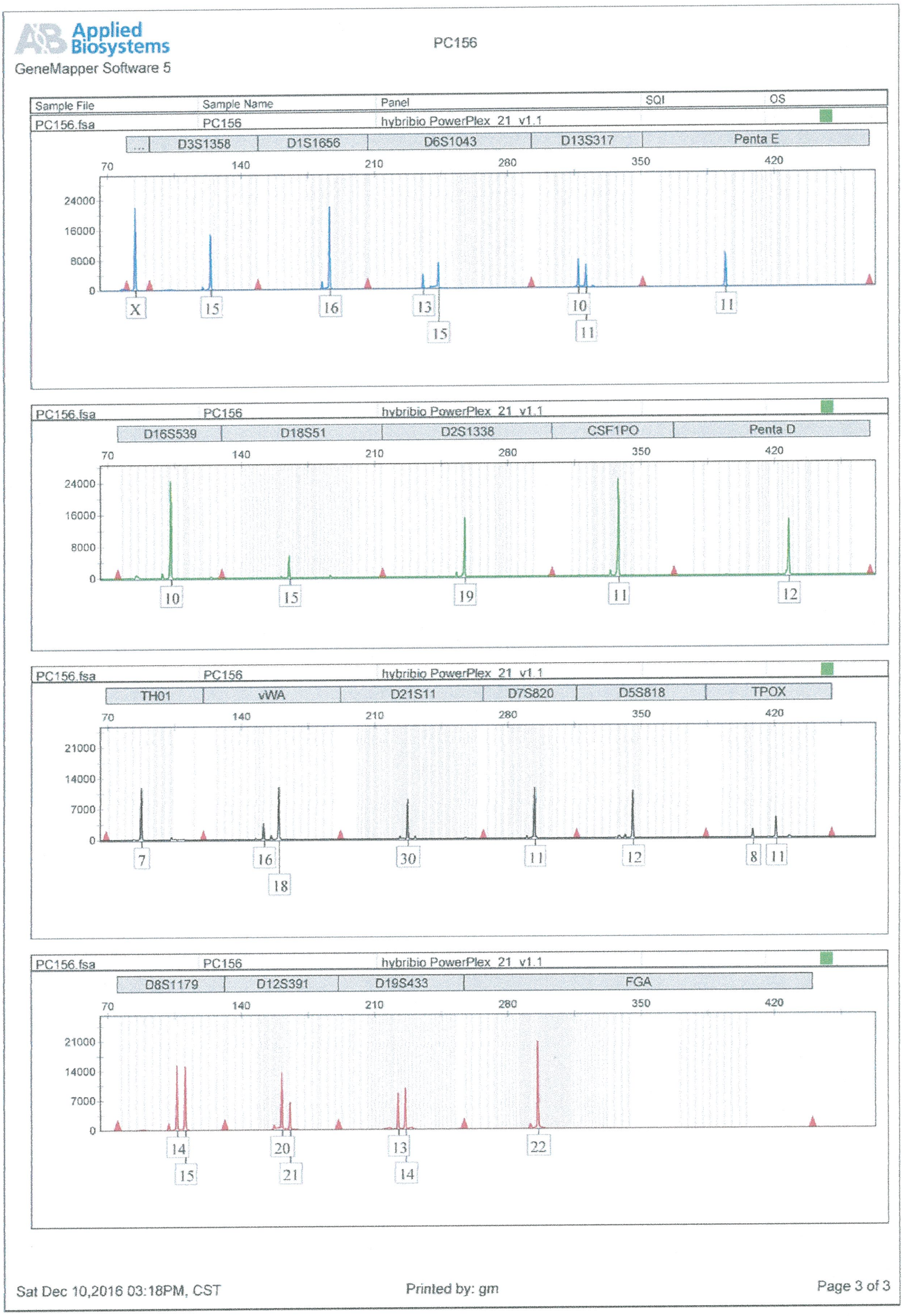

Supplement: Supplementary file 1 — Additional file 1. [file 40360_2020_461_MOESM1_ESM.pdf]
